# Supplementary material for: Impacts of COVID-19 on sexual behaviors, HIV prevention and care among men who have sex with men: A comparison of New York City and Metropolitan Atlanta
Source: PLoS One. 2023 Mar 21;18(3):e0282503. doi: 10.1371/journal.pone.0282503 (PMC10030006; doi:10.1371/journal.pone.0282503)
Supplement: S1 File — (DOCX) [file pone.0282503.s001.docx]

**Impacts of COVID-19 on sexual behaviors, HIV prevention and care among men who have sex with men: a comparison of New York City and Metropolitan Atlanta**

Online Supplement

**Table of Contents**

[**Extraction of test data** 2](#_Toc128409946)

[**Note on AMIS surveys** 2](#_Toc128409947)

[**PrEP prescriptions (Data source: IQVIA)** 3](#_Toc128409948)

[Figure S1: Interrupted time series analysis for number of men with active PrEP prescriptions 3](#_Toc128409949)

[Table S1: Interrupted time series model parameters for number of men with active PrEP prescriptions 3](#_Toc128409950)

[**ART prescriptions (Data source: IQVIA)** 4](#_Toc128409951)

[Figure S2: Interrupted time series analysis for number of men with active ART prescriptions 4](#_Toc128409952)

[Table S2: Interrupted time series model parameters for number of men with active ART prescriptions 4](#_Toc128409953)

[Figure S3: Prevalence of active ART prescriptions by age, relative to the weekly mean Feb. 2-Feb. 29, 2020. 5](#_Toc128409954)

[**HIV tests (Data source: Labcorp and Quest Diagnostics)** 6](#_Toc128409955)

[Figure S4: Interrupted time series analysis for number of valid HIV tests on males 6](#_Toc128409956)

[Table S3: Interrupted time series model parameters for number of valid HIV tests on males 6](#_Toc128409957)

[Figure S5: Prevalence of HIV tests by age, relative to the weekly mean Feb. 2-Feb. 29, 2020. 7](#_Toc128409958)

[**Sexual partner counts (Data source: AMIS)** 8](#_Toc128409959)

[Figure S6: Number of anal intercourse (AI) partners in the last 12 months, by survey, city and age 8](#_Toc128409960)

[**Predictors of reporting that COVID-19 led to a decrease in sexual partners (Data source: AMIS)** 9](#_Toc128409961)

[Table S4: Multivariate models predicting whether a respondent says that the COVID-19 pandemic caused them to decrease their number of sexual partners 9](#_Toc128409962)

# **Extraction of test data**

From each of our two laboratory data sources (Labcorp and Quest Diagnostics), we counted all test orders with either an order or result containing “56888-1” within the reported Logical Observation Identifiers Names and Codes (LOINC®, https://loinc.org/56888-1). This LOINC is indicative of an HIV-1/HIV-2 Antigen Antibody test recommended for use as the HIV screening test since 2014, and is a component of nearly all panels that included HIV testing.

# **Note on AMIS surveys**

In additional to the regular annual AMIS surveys, AMIS staff also developed two smaller flash surveys in April and July of 2020 in response to COVID-19, which we do not analyze given the very small sample sizes for specific geographic areas. Also, in each round of the regular survey, roughly 10% of the sample is drawn from the previous year’s respondent pool, providing opportunities for longitudinal analysis. However, given our geographical restrictions, the number of respondents appearing in both our survey years was small (39 in Atlanta, 26 in NYC), such that we did not conduct longitudinal analyses on them specifically.

# **PrEP prescriptions (Data source: IQVIA)**

## Figure S1: Interrupted time series analysis for number of men with active PrEP prescriptions


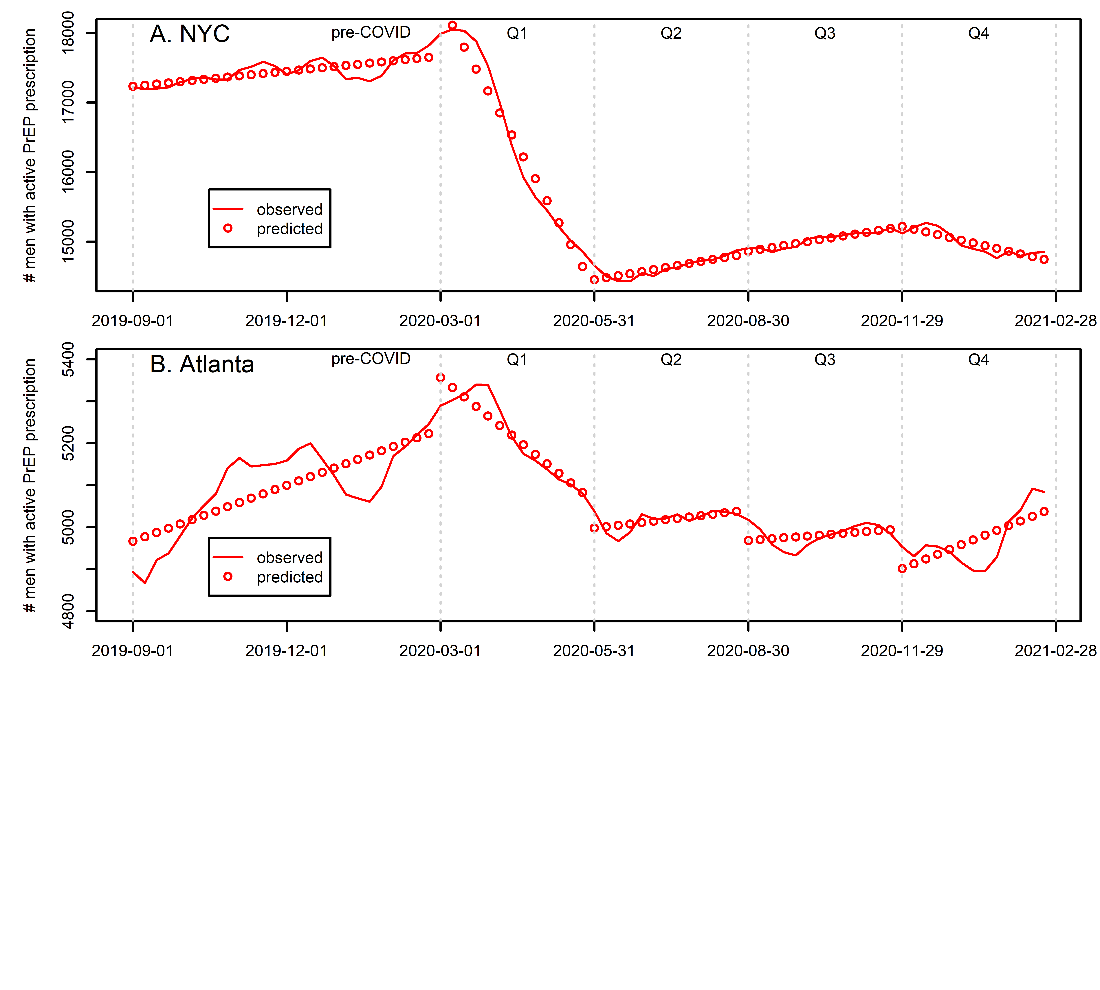


## Table S1: Interrupted time series model parameters for number of men with active PrEP prescriptions

|  | NYC | |  | Atlanta | |
| --- | --- | --- | --- | --- | --- |
| *R^2^* | 0.990 | |  | 0.831 | |
|  | coef. | P-value |  | coef. | P-value |
| Intercept | **17215.3** | **<0.01** |  | **4956.6** | **<0.01** |
| Week | **16.7** | **<0.01** |  | **10.3** | **<0.01** |
| Q1 | **9727.4** | **<0.01** |  | **1014.8** | **<0.01** |
| Q2 | **-3936.8** | **<0.01** |  | -90.4 | 0.60 |
| Q3 | **-3803.5** | **<0.01** |  | -100.8 | 0.65 |
| Q4 | 612.0 | 0.42 |  | **-802.2** | **<0.01** |
| Q1:week | **-332.1** | **<0.01** |  | **-33.0** | **<0.01** |
| Q2:week | 12.6 | 0.26 |  | -7.0 | 0.08 |
| Q3:week | 10.7 | 0.34 |  | **-8.1** | **0.04** |
| Q4:week | **-56.2** | **<0.01** |  | 1.1 | 0.79 |

# **ART prescriptions (Data source: IQVIA)**

## Figure S2: Interrupted time series analysis for number of men with active ART prescriptions


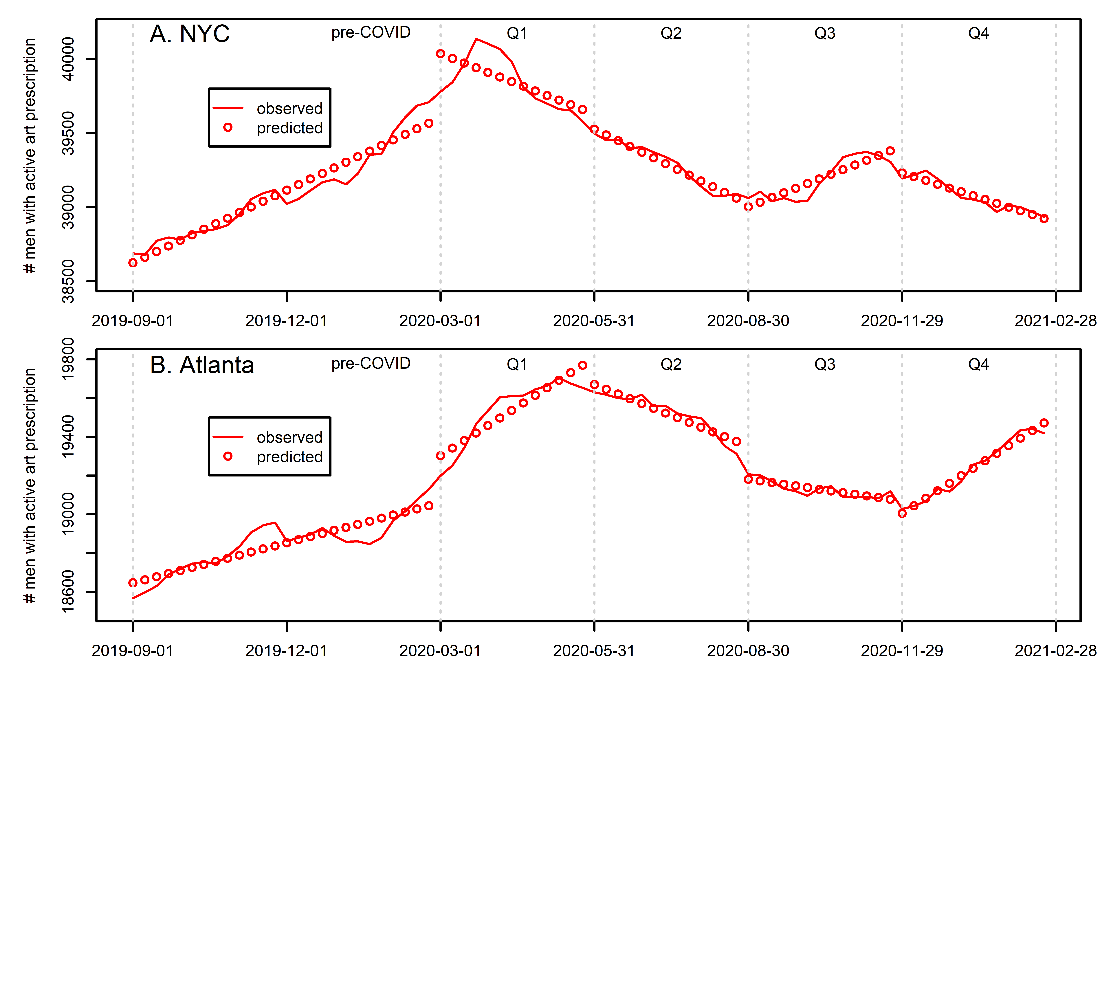


## Table S2: Interrupted time series model parameters for number of men with active ART prescriptions

|  | NYC | |  | Atlanta | |
| --- | --- | --- | --- | --- | --- |
| R^2^ | 0.947 | |  | 0.973 | |
|  | coef. | P-value |  | coef. | P-value |
| Intercept | **38587** | **<0.01** |  | **18630.2** | **<0.01** |
| Week | **37.7** | **<0.01** |  | **15.9** | **<0.01** |
| Q1 | **2291.5** | **<0.01** |  | **-378.4** | **<0.01** |
| Q2 | **2492.3** | **<0.01** |  | **2017.3** | **<0.01** |
| Q3 | **-1251.5** | **<0.01** |  | **1009.1** | **<0.01** |
| Q4 | **2341.1** | **<0.01** |  | **-2188.6** | **<0.01** |
| Q1:week | **-68.9** | **<0.01** |  | **23.0** | **<0.01** |
| Q2:week | **-76.5** | **<0.01** |  | **-40.4** | **<0.01** |
| Q3:week | -6.2 | 0.35 |  | **-24.6** | **<0.01** |
| Q4:week | **-63.4** | **<0.01** |  | **22.9** | **<0.01** |

## Figure S3: Prevalence of active ART prescriptions by age, relative to the weekly mean Feb. 2-Feb. 29, 2020.

A) New York City B) Metro Atlanta


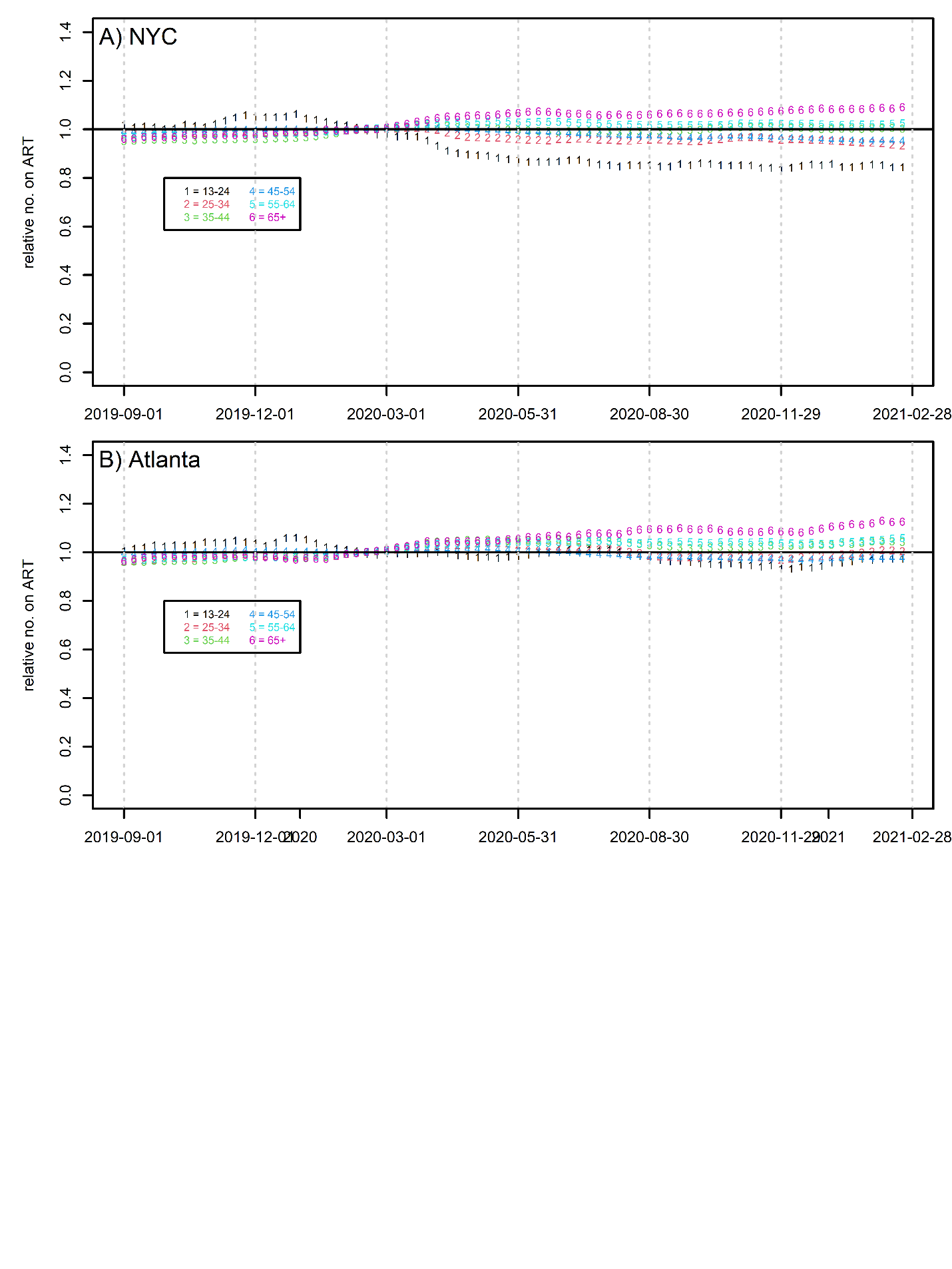


# **HIV tests (Data source: Labcorp and Quest Diagnostics)**

## Figure S4: Interrupted time series analysis for number of valid HIV tests on males


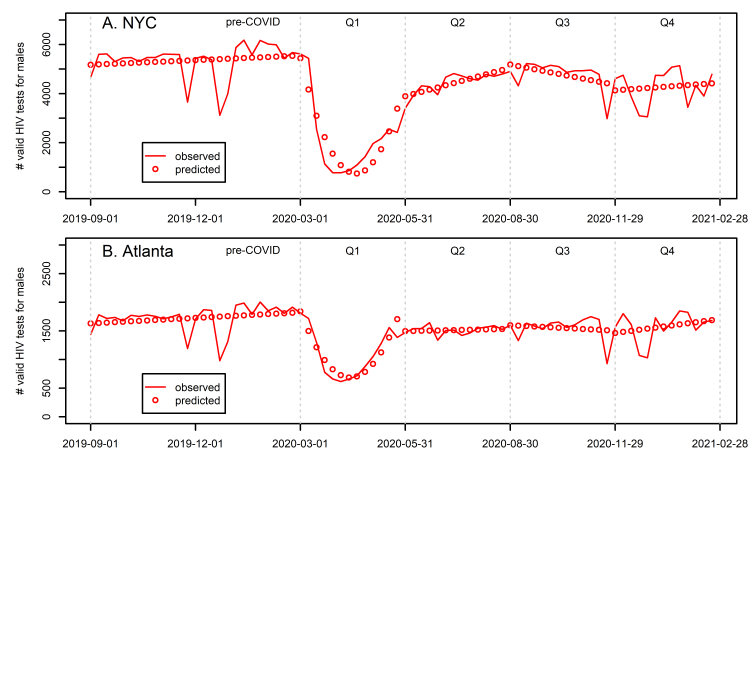


## Table S3: Interrupted time series model parameters for number of valid HIV tests on males

|  | NYC | |  | Atlanta | |
| --- | --- | --- | --- | --- | --- |
| R^2^ | 0.798 | |  | 0.637 | |
|  | coef. | P-value |  | coef. | P-value |
| Intercept | **5,160.0** | **<0.01** |  | **1,622.2** | **<0.01** |
| Week | 14.4 | 0.41 |  | 7.4 | 0.19 |
| Q1 | **110,220.9** | **<0.01** |  | **32,338.2** | **<0.01** |
| Q2 | **-4,831.7** | **0.04** |  | -247.7 | 0.74 |
| Q3 | 3,396.4 | 0.25 |  | 363.1 | 0.70 |
| Q4 | -2,576.1 | 0.47 |  | -1,398.1 | 0.22 |
| Q1:week | **-6,787.1** | **<0.01** |  | **-2,012.8** | **<0.01** |
| Q1:week^2^ | **100.0** | **<0.01** |  | **30.2** | **<0.01** |
| Q2:week | 74.7 | 0.16 |  | -4.4 | 0.79 |
| Q3:week | -78.1 | 0.14 |  | -14.7 | 0.38 |
| Q4:week | 9.0 | 0.86 |  | 11.3 | 0.50 |

## Figure S5: Prevalence of HIV tests by age, relative to the weekly mean Feb. 2-Feb. 29, 2020.

A) New York City, B) Metro Atlanta


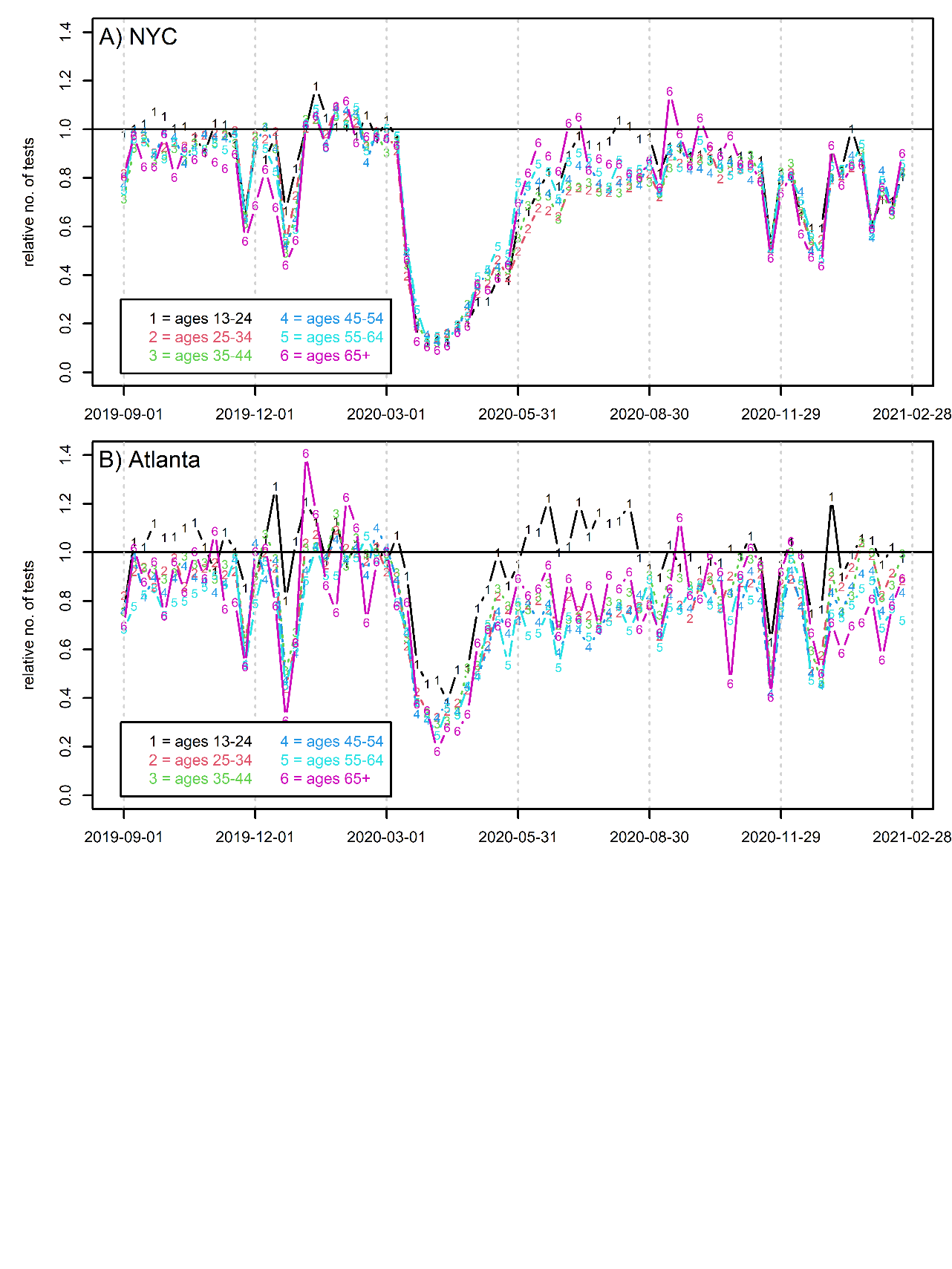


# **Sexual partner counts (Data source: AMIS)**

## Figure S6: Number of anal intercourse (AI) partners in the last 12 months, by survey, city and age


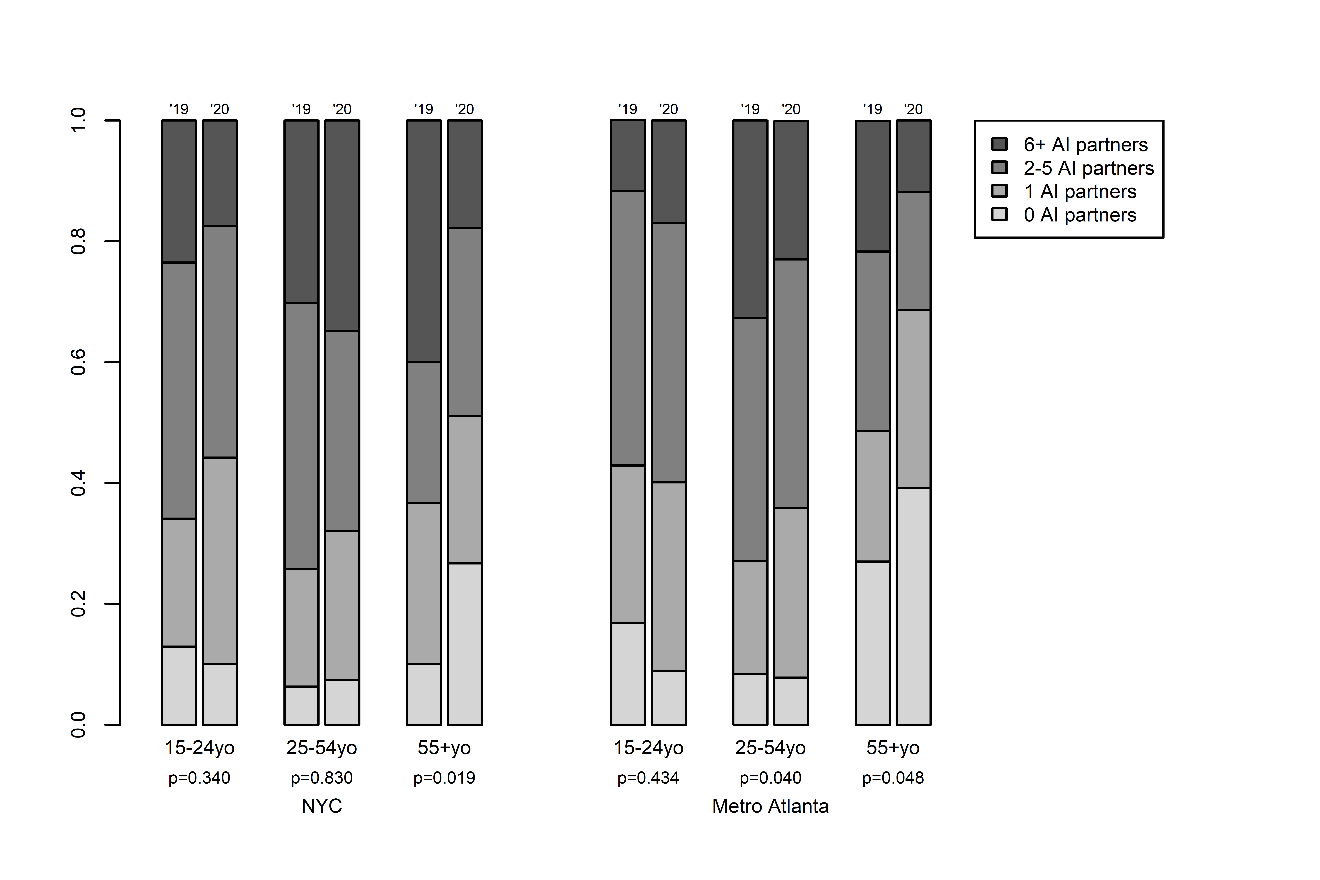


Left-hand columns per pair = data from the 2019 round of AMIS

Right-hand columns per pair = data from the 2020 round of AMIS

# **Predictors of reporting that COVID-19 led to a decrease in sexual partners (Data source: AMIS)**

## Table S4: Multivariate models predicting whether a respondent says that the COVID-19 pandemic caused them to decrease their number of sexual partners

|  |  | NYC | |  | Metro Atlanta | |
| --- | --- | --- | --- | --- | --- | --- |
|  |  | OR | P-value |  | OR | P-value |
| **Age** |  |  |  |  |  |  |
| 15-24 |  | 0.45 | 0.06 |  | 1.10 | 0.82 |
| 25-54 |  | referent | |  | referent | |
| 55+ |  | 1.75 | 0.24 |  | **2.46** | **0.04** |
| **Race/ethnicity** |  |  |  |  |  |  |
| Black (non-Hisp.) |  | **0.30** | **0.01** |  | 0.88 | 0.74 |
| Hispanic |  | 1.04 | 0.93 |  | 0.24 | 0.20 |
| White (non-Hisp.) |  | referent | |  | referent | |
| Another race (non-Hisp.) |  | 1.35 | 0.61 |  | 0.91 | 0.87 |
| **Living with HIV diagnosis** |  | 0.98 | 0.97 |  | 1.27 | 0.60 |
| **Eligible for PrEP** |  | 1.67 | 0.16 |  | 1.46 | 0.34 |
| **Used PrEP in last 12 mos.** |  | 2.08 | 0.06 |  | 1.71 | 0.24 |
| **Living with partner** |  | 0.56 | 0.13 |  | **0.33** | **0.01** |
| **More than HS education** |  | 0.38 | 0.20 |  | 1.65 | 0.32 |
| **Annual HH income > $40k** |  | 0.93 | 0.85 |  | 1.17 | 0.65 |
