# Supplementary material for: Impacts of COVID-19 on sexual behaviors, HIV prevention and care among men who have sex with men: A comparison of New York City and Metropolitan Atlanta
Source: PLoS One. 2023 Mar 21;18(3):e0282503. doi: 10.1371/journal.pone.0282503 (PMC10030006; doi:10.1371/journal.pone.0282503)
Supplement: Attachment — Submitted filename: COVID-HIV NYC-ATL_response_PLoS_One_resub.docx [file pone.0282503.s002.docx]

**We thank the editors and the reviewers for the very positive reviews, and minimal comments. Our responses are below:**

Reviewer #1: Overall, this is a wonderfully conducted and well-written paper. I usually have a raft of comments but this rare case, I don't have anything to add. Great job on this, it was a pleasure to read and will be a welcome contribution to the literature.

**Thank you very much!**

Reviewer #2: This was an excellent paper modeling the impact of the COVID-19 pandemic and subsequent shelter-in-place ordinances on HIV-related outcomes for men who have sex with men in Atlanta and New York City. The paper was richly and exhaustively detailed, included robust analyses, and provided detailed interpretations of what the findings mean. I commend the authors on an excellent analysis and write-up. I have only a couple minor comments and suggestions:

**Thank you very much!**

I recommend the authors relocate their discussion of the ITS methods from their supplement to the primary paper. Given that interrupted time series analyses are an extension of other longitudinal statistical modeling approaches, it would be helpful for the authors to include this description in the primary paper for the readership.

**We have moved the explanation of the ITS methods from the supplement to the main paper and integrated it there. We agree this now makes the component of the paper clearer. We believe the reviewer was only referring to the initial description of the methods here, and not also to the presentation of results and their interpretation, given that this would increase the number of figures in the paper to at least 9. If we misunderstood and the reviewer or editor wants more material moved, we are happy to do so.**

In the Results for AMIS, the authors report that substantial fractions of men in Atlanta and NYC self-reported declines in partner numbers as a result of the COVID-19 pandemic. It would be helpful for the authors to present (even unadjusted) characteristics associated with partner reductions, which could help guide inferences regarding whether those who were at greatest risk for HIV reduced partners during the pandemic and would complement their other findings well.

**Thank you for suggesting this. This was among the many additional analyses we considered, but struggled with knowing when to stop given the richness of the data sources. We have now added it in to the paper. We opted to put the table itself in the supplement, given the large number of tables and figures already, but put the presentation of results and discussion in the main manuscript. We are happy to move the table to the main manuscript if the editor prefers that.**
